# Validity of visceral adiposity estimates from DXA against MRI in Kuwaiti men and women

**DOI:** 10.1038/nutd.2016.38

**Published:** 2017-01-09

**Authors:** A Mohammad, E De Lucia Rolfe, A Sleigh, T Kivisild, K Behbehani, N J Wareham, S Brage, T Mohammad

**Affiliations:** 1 Department of Public Health Research, Dasman Diabetes Institute, Kuwait City, Kuwait; 2 Medical Research Council Epidemiology Unit, University of Cambridge School of Clinical Medicine, Institute of Metabolic Science, Cambridge Biomedical Campus, Cambridge, UK; 3 Wolfson Brain Imaging Centre, University of Cambridge School of Clinical Medicine, and NIHR/Wellcome Trust Clinical Research Facility, Cambridge University Hospitals NHS Foundation Trust, Cambridge Biomedical Campus, Cambridge, UK; 4 Department of Biological Anthropology, University of Cambridge, Cambridge, UK

## Abstract

**Objectives:**

The prevalence of obesity and diabetes in the Middle East is among the highest in the world. Valid measures of abdominal adiposity are essential to understanding the metabolic consequences of obesity. Dual-energy X-ray absorptiometry (DXA) is increasingly being utilised to assess body composition in population studies, and has recently been used to estimate visceral adipose tissue (VAT). The aim of this study was to determine the accuracy of DXA-derived VAT in a Middle Eastern population using magnetic resonance imaging (MRI) as the criterion measure.

**Method:**

VAT was estimated from abdominal DXA measures in 237 adult men (*n*=130) and women (*n*=107), aged 18–65 years, participating in the Kuwait Wellbeing Study. These estimates were compared with MRI measures of the corresponding anatomical region. The agreement between methods was assessed using Bland–Altman as well as correlation analysis.

**Results:**

Median MRI VAT was 1148.5 cm^3^ (95% confidence interval: 594.2–1734.6) in men and 711.3 cm^3^ (95% confidence interval: 395.5–1042.8) in women. DXA estimates of VAT showed high correlations with corresponding MRI measures (*r*=0.94 (*P*<0.0001) in men; *r*=0.93 (*P*<0.0001) in women). DXA overestimated VAT with a mean bias (95% limits of agreement) of 79.7 cm^3^ (−767 to 963) in men and 46.8 cm^3^ (−482 to 866) in women. The imprecision of DXA increased with increasing VAT level in both men and women.

**Conclusion:**

DXA estimates of VAT are valid for use in Middle Eastern populations, although accuracy decreases with increasing level of visceral adiposity.

## Introduction

Obesity and its related comorbidities have become a major public health challenge in the Middle East and North Africa (MENA) region.^1, 2, 3, 4^ Data published by the International Diabetes Federation have shown that the MENA region has the highest prevalence of diabetes in the world, and the country of Kuwait is ranking top in this region at 20.7%.^5^ Obesity is particularly prevalent in this country, where 59% of women and 46% of men are either overweight or obese.^6^ Lifestyle changes, such as decreasing levels of physical activity and increased consumption of energy-dense diets, resulting from rapid urbanisation and economic growth over recent decades, are possible contributing factors to this high prevalence.^7^

Abdominal obesity, and in particular increased visceral adipose tissue (VAT), is associated with insulin resistance, glucose intolerance and type 2 diabetes.^8, 9, 10^ VAT is considered to be more sensitive to lipolytic stimuli than other adipose depots,^11, 12^ and it has been suggested that visceral obesity decreases insulin action via increased delivery of free fatty acids in insulin-sensitive tissues.^13, 14^ It is therefore plausible that VAT is implicated in the pathophysiology of type 2 diabetes, the investigation of which relies on accurate quantification of VAT. However, limited data are available on VAT and how this parameter relates to overall obesity and diabetes in populations in the MENA region.

Direct imaging techniques such as computerised tomography (CT) and magnetic resonance imaging (MRI) are the reference methods used for the quantification of VAT. Their use in large-scale population studies is, however, limited because of cost, lengthy data post processing and the exposure to ionising radiation in the case of CT.^15, 16^ Consequently, only standard anthropometry measures, which lack the ability to distinguish between visceral and subcutaneous adipose compartments, are typically used in such studies. Dual-energy X-ray absorptiometry (DXA) is increasingly being implemented in large-scale epidemiological studies to assess overall and regional body composition. The recent introduction of DXA instruments that provide an estimate of VAT from the abdominal DXA scan has opened potential avenues to the measurement of VAT in large-scale population studies.^15^ However, the algorithms involved in the process of quantifying VAT from DXA are not publically available and verification of this measure has only been investigated in American and Asian Chinese adults.^15, 17, 18^ The aim of this study was to validate the estimation of DXA-derived VAT using MRI-measured VAT as the criterion method in Kuwaiti men and women aged 18 to 65 years. This DXA method could aid large-scale studies investigating VAT in MENA region populations that may help elucidate the interplay between this fat compartment and the metabolic consequences associated with obesity.

## Materials and methods

### Study participants

The study included a total of 237 adult men (*n*=130) and woman (*n*=107) between the ages of 18 and 65 years who took part in the Kuwait Wellbeing Study. Participants attended the Kuwait Wellbeing Unit at Dasman Diabetes Institute between November 2012 and April 2013. The exclusion criteria included pregnancy, known diabetes, standard MRI contradictions, inability to walk unaided, psychosis or terminal illness. All measurements on each individual were performed on the same day.

This study was approved by the Ethical Review Board of the Dasman Diabetes Institute, Safat, Kuwait, and all participants provided informed signed consent.

### Anthropometric measurements

Anthropometry was performed by trained nurses. Weight was measured using a calibrated scale (TANITA model BC-418 MA, Tokyo, Japan) and recorded to the nearest 0.1 kg. Height was measured by a wall mounted stadiometer (SECA model 240, Birmingham, UK) and recorded to the nearest 0.1 cm. Body mass index was calculated as weight/height^2^ (kg m^−2^). Waist and hip circumferences were measured with a D-loop tape measure at the mid-point between the lowest rib margin and the iliac crest and the widest level over the greater trochanters respectively. Both measurements were recorded to the nearest 0.1 cm. The participants were fasted and wearing loose clothing during the measurements.

### DXA measurements

**Figure 1:**
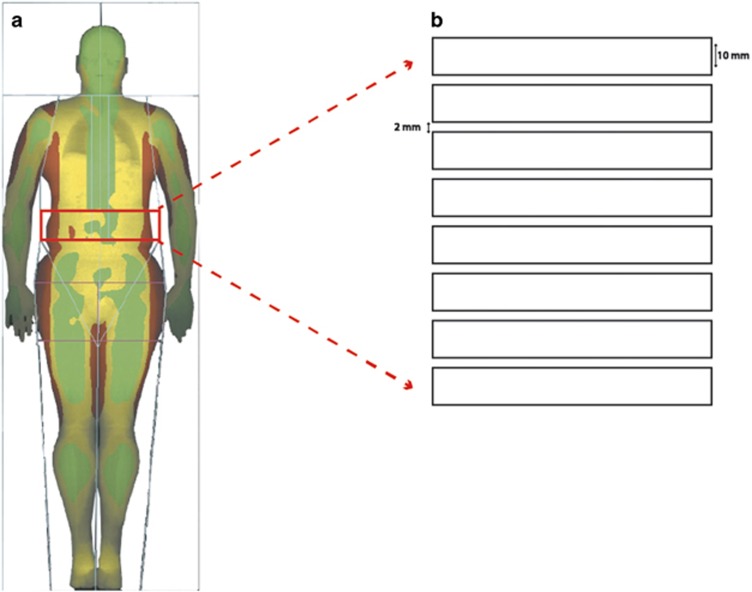
(**a**) Image of a full-body DXA scan. The red box depicts the region automatically outlined by iliac crest and with a superior height equivalent to 20% of the distance from the top of the iliac crest to the base of the skull, used to calculate VAT. (**b**) An illustration of the MRI slices (10 mm) and interslice gaps (2 mm) that match the region used by the DXA method to measure VAT.

Total body imaging was acquired using the Lunar iDXA (GE Healthcare, Bedford, UK). Estimates of total body fat mass, android fat mass and VAT content (mass and volume) were derived using the iDXA enCORE software (version 14.10.022; GE Heathcare). The software estimates the VAT content within the android region; the software automatically places a quadrilateral box, that is, the region outlined by the iliac crest and with a superior height equivalent to 20% of the distance from the top of the iliac crest to the base of the skull^17^ (Figure 1a).

Individuals who were broader than the scanning area (*n*=80) were positioned for either a sagittal half-body scan (the left side of the body) or placed to exclude the right arm in the field of view. Body composition variables were then recalculated in those participants to include the missing regions by assuming left–right body symmetry.

Daily quality assurance and quality controls were carried out during the study period before using the equipment according to standard procedures supplied by the manufacturer. The volunteers were scanned using standard imaging and positioning protocols^19, 20^ by three trained operators. All the images were processed by one trained researcher (AM).

### MRI measurements

The MRI images were acquired by trained radiographers. The participants were placed supine in a GE 3T Discovery 750 whole body scanner. A 32-channel torso coil was used to acquire 21 FSE-IDEAL transaxial slices with respiratory gating, centred on the L4 vertebral level. The in-plane resolution was 0.94 × 0.94 mm, field of view 480 × 480 mm, slice thickness 10 mm with an interslice gap of 2 mm. The GE reconstructed fat images from the IDEAL sequence were used to calculate the volume of VAT using the software Analyze 11.0 (BIR, Mayo Clinic, Rochester, MN, USA) as described previously.

MRI VAT volumes were calculated from the same android region defined by the DXA to enable an accurate comparison between MRI and DXA estimates of VAT. This was achieved on an individual basis by defining the MRI slices to match the region used by the DXA method (see DXA methods). The MRI VAT area from each of these slices was multiplied by 12 mm (the interslice distance, Figure 1b) to convert it to a volume before summation to yield an MRI VAT that is comparable to DXA VAT. If the DXA region did not match an integer number of MRI slices, the contribution from the final MRI slice was weighted accordingly. The fraction of the final slice (last slice factor), was defined as follows: Last slice factor=[0.2A−(10 NS+2 (NS−1))]/11, where *A* is abdomen length in mm and NS is number of full slices.

An estimate of total abdominal fat (TAF) was also calculated to compare it against DXA-derived TAF. TAF was defined as VAT+SCAT, where SCAT is the subcutaneous adipose tissue measured from the same abdominal region as the VAT. The images were processed and analysed by one researcher.

### Statistical analysis

Statistical analyses were performed using STATA (version 12; StataCorp, College Station, TX, USA). Statistical significance was set at *P*<0.05. Results are reported as mean±s.d. or if the variables were not normally distributed as median and interquartile ranges. Unpaired *t-*test and Kruskal–Wallis tests were used to compare sample characteristics by sex. Spearman's rank coefficients were calculated to investigate correlations between MRI criterion measures and different measures of abdominal fat.

Linear regression analysis was used to quantify the proportion of variance of VAT measured by MRI explained by DXA and anthropometry.

The level of agreement in VAT and TAF between DXA and MRI was determined using Bland–Altman plots, with mean difference (bias) tested using paired *t*-tests. A regression line was added to the Bland–Altman plots indicating error trend throughout the measurement range. Limits of agreement were calculated as bias±1.96 s.d. of the difference, and heteroscedasticity was determined using correlation of absolute differences against the MRI criterion. Non-normally distributed variables were log transformed before analysis.

## Results

The characteristics of the study participants are shown in Table 1. Men were taller, had larger waist circumference and waist to hip ratio than women and had more VAT (DXA and MRI).

### Relative validity

Table 2 shows Spearman's correlation coefficients between anthropometry and DXA measures and abdominal adiposity volumes (total and VAT) as measured by MRI (the criterion method) in men and women separately. VAT volume from DXA was most strongly related to VAT from MRI in men (*r*=0.94) and women (*r*=0.93). Of the anthropometry measures, body mass index and waist circumference showed the strongest correlations with VAT (*r*=0.72 to *r*=0.77). A multiple regression model including weight, waist and hip circumference explained 56–59% of the variance in MRI-measured VAT, whereas DXA-measured VAT volume explained 80% and 86% of the variance in MRI in women and men, respectively. Adding all predictors to the same model rendered only the DXA variable significant.

### Absolute validity

**Figure 2:**
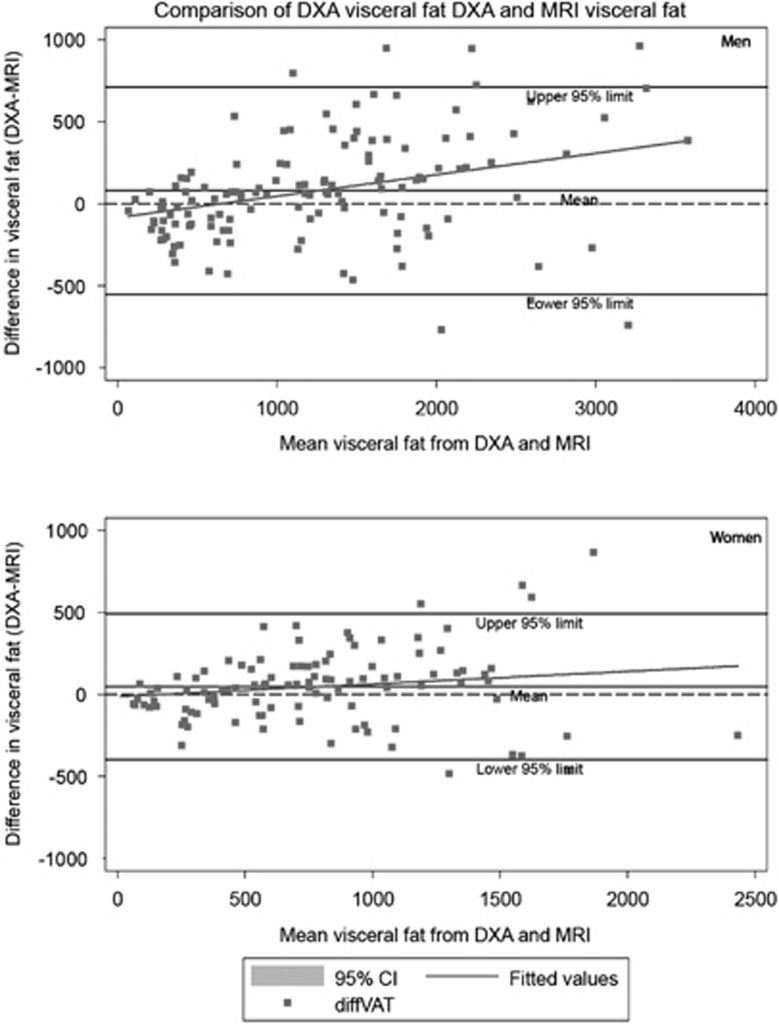
Bland–Altman analysis for men (upper panel) and women (lower panel) comparing the difference between visceral adipose tissue (cm^3^) from DXA and visceral adipose tissue (cm^3^) from MRI with the mean visceral fat from the two methods; the Kuwait Wellbeing Study 2012–2013 (*n*=237).

**Figure 3:**
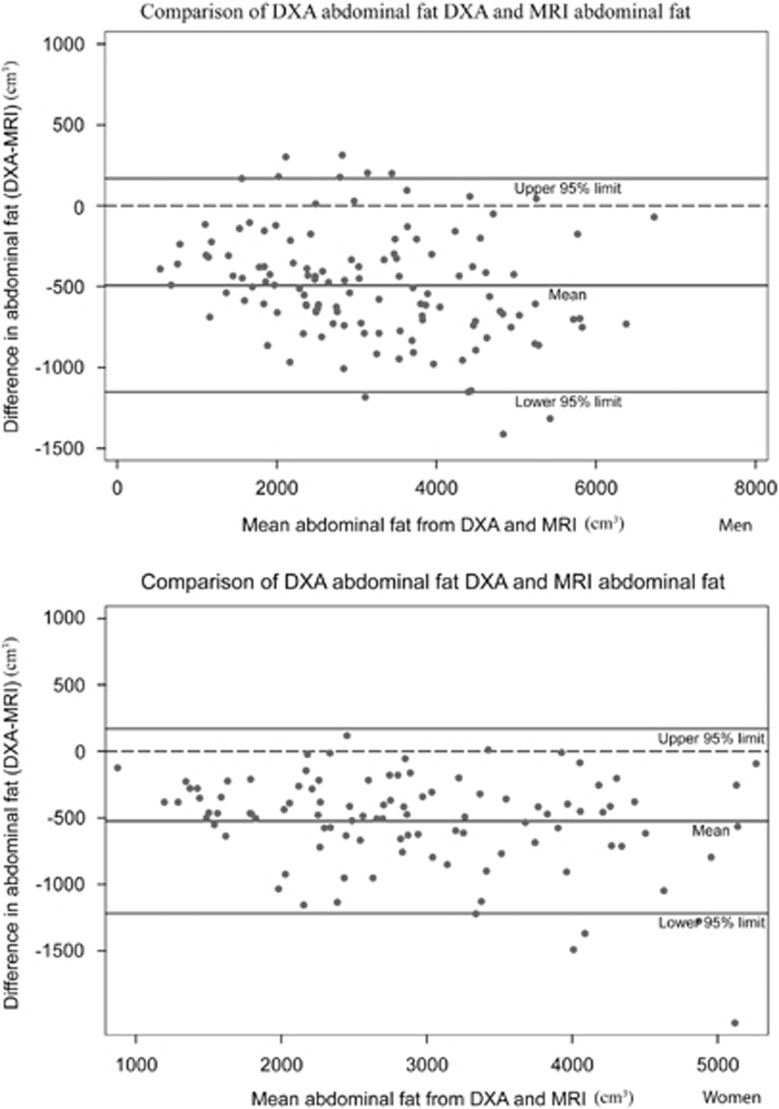
Bland–Altman analysis for men (upper panel) and women (lower panel) comparing the difference between abdominal fat mass (cm^3^) from DXA and abdominal fat mass (cm^3^) from MRI with the mean abdominal fat from the two methods; the Kuwait Wellbeing Study 2012–2013 (*n*=237).

DXA overestimated VAT by roughly 7% in both men and women; the mean biases (95% limits of agreement) were 79.7 cm^3^ (−565.3 to 724.7; *P*=0.006) in men and 46.8 cm^3^ (−406.4 to 498.5; *P*=0.04) in women. As can be seen in the Bland–Altman plots (Figure 2), there was evidence of heteroscedasticity, that is, increasing scatter with increasing VAT, in both men (*r*=0.94, *P*⩽0.00010) and women (*r*=0.93, *P*⩽0.0001). In contrast to the VAT results, DXA significantly underestimated TAF compared with MRI in men and women (Figure 3). The mean difference was −492.5 cm^3^ (−1165.5 to 180.5) in men (*P*<0.0001) and −522.9 cm^3^ (−1229 to 185) in women (*P*<0.0001) or roughly 16% of the mean values. Estimation errors did not differ significantly by sex (*P*=0.32).

## Discussion

In this study, we assessed the validity of DXA-derived VAT estimates in a cohort of Kuwaiti men and women using MRI as the criterion method. The results showed a small positive bias of ∼7% for DXA-based VAT estimates, but high correlations in both men and women, associations that were much stronger than those found for anthropometric measures.

These results provide justification to using DXA as an alternative to standard epidemiological estimation techniques of abdominal obesity such as waist and hip circumference, but likely only in the scenario where DXA was included in the study design for its primary purpose of measuring whole-body composition. Clearly, the feasibility of DXA is much lower than for example a waist circumference measurement, but if the X-ray images are acquired anyway, our results indicate that more accurate estimates of VAT can be obtained. Despite the strong associations, DXA overestimated VAT, particularly in individuals with higher content of VAT; the overestimation was more apparent in participants with VAT of >750 cm^3^ for women and 1500 cm^3^ for men. That being said, individuals with the very lowest VAT levels were underestimated by DXA, as indicated by the error trend line in the Bland–Altman plots.

Our findings concur with other studies in adult men and women of different ethnicity. A validation study carried out in Chinese adults using CT as criterion also found a significant overestimation of DXA-derived VAT,^18^ with biases of 143 cm^3^ for women and 379 cm^3^ in men and 95% limits of agreement of −232 to 755 cm^3^ for both sexes combined.

Strong correlations were also observed in a Chinese population reporting correlations of 0.947 for women and 0.891 for men.^18^ Even stronger correlations were reported for an American population, ranging from 0.959 in women to 0.949 in men.^17^

Our study population had similar DXA-derived VAT volumes (1232 (516.3–1634.6) in men and 789.4 (410.2–1090) in women) compared with the sample of American adults who were predominantly Caucasian; the mean DXA VAT volumes in that study were 1382±945 (men) and 800±960 (women).^17^ In contrast, the mean DXA-derived VAT in this adult Kuwaiti cohort was lower than that observed in the Chinese population, with values ranging between 1560±587 (men) and 1000±715 (women). The higher mean observed in the Chinese study may be a consequence of the older average age of the Chinese participants (~51 years) compared with the Kuwaiti participants (~40 years), as visceral adiposity increases with increasing age.^18^

The overestimation of VAT observed in this and other studies may be explained by DXA relying on differential attenuation of two X-rays of different wavelengths; consequently, in larger individuals where the X-rays of both wavelengths will be more attenuated, the signal-to-noise ratio between them, and hence the ability to differentiate tissues, will be diminished.^21^ Another explanation for the discrepancy may be due to the fact that fat is also found in muscle, solid organs and bones. Although these fat compartments are much smaller, all fat is captured by the DXA method, but the fat from these other compartments is removed in MRI method when assessing VAT.^22^ Abdominal fat measured by DXA showed strong correlations with MRI in both men (*r*=0.97) and women (*r*=0.95). However, when compared with MRI, DXA significantly underestimated this compartment in both men and women, findings that are concordant with other studies. In these studies, the investigators defined subregions on the DXA that corresponded to the CT anatomical abdominal area and showed that DXA underestimation ranged from 10 to 26%.^23, 24, 25^ The underestimation might be a result of the variation in fat content and differences in the relative amounts of fat in subcutaneous and visceral adipose tissue.^24^

The main strengths of our study were the large sample size, covering a wide range of adiposity, and the inclusion of an equal number of men and women with an evenly distributed age range. The main limitations of the study include potential mismatch of the anatomical areas that were assessed by the two methods. The DXA method uses 20% of the length of the abdomen from the iliac crest, and although the MRI slice region was chosen and processed to cover the same as the DXA, there may still be a small degree of non-overlap of the regions. However, previous work only used a set of number of slices.^17^

In conclusion, DXA estimates of VAT have a small positive bias, but are valid for ranking individuals with higher or lower visceral fat volumes and may be considered an alternative assessment to employ when reference methods like MRI and CT are not feasible. Although the DXA method is poor at determining absolute values of VAT volumes at the individual level, this approach for estimating VAT may aid the study of the mechanisms involved in metabolic regulation and the effects of different abdominal fat depots on the risk of developing obesity-related disorders in the MENA region.

## Figures and Tables

**Table 1:** Characteristics of the study sample; the Kuwait Wellbeing Study 2012–2013 (*n*=237)

*Median (interquartile range)*	*Men*	*Women*	P*-value*^a^
	(n=*130)*	(n=*107)*	
Age (years)	38.8±10.4	43.1±11.3	0.003
			
*Anthropometric measures*			
Weight (kg)	83.1 (74.3–95.6)	73.6 (64.1–85.3)	<0.0001
Height (cm)	173.3±6.6	159.4±5.4	<0.0001
BMI (kg m^−2^)	28.4±4.6	29.6±6.1	0.09
			
*MRI measures*			
VAT (cm^3^)	1148.5 (594.2–1734.6)	711.3 (395.5–1042.8)	0.0001
Abdominal fat (cm^3^)	3217.9 (2385.5–4392.8)	2958.5 (2359.6–3959.8)	0.25
			
*DXA measures*			
VAT (cm^3^)	1232.6 (516.3–1852)	789.4 (410.2–1090)	<0.0001
VAT (kg)	1.2 (0.5–1.7)	0.7 (0.387–1.0)	<0.0001
Abdominal fat (cm^3^)	2797.7 (2032.6–3829)	2559.3 (1930–3408)	0.15
Body fat %	32±6.9	44.3±5.5	<0.0001

Abbreviations: BMI, body mass index; DXA, dual-energy X-ray absorptiometry; MRI, magnetic resonance imaging; VAT, visceral adipose tissue.

Data are presented as mean±s.d. or median (interquartile ranges).

a Sex differences by *t*-test or Kruskal–Wallis rank test. DXA AF is derived variable from DXA android fat–DXA-estimated VAT.

**Table 2:** Spearman's rank correlation coefficients between anthropometry DXA and MRI measures of abdominal adiposity in both men and women; the Kuwait Wellbeing Study 2012–2013 (*n*=237)

		*MRI measures*			
		*Men*		*Women*	
		*VAT (cm*^*3*^)	*AF*^a^ *(cm*^*3*^)	*VAT (cm*^*3*^)	*AF (cm*^*3*^)
					
*Anthropometry* Weight (kg)		0.69	0.82	0.7	0.87
BMI (kg m^−2^)		0.74	0.85	0.73	0.87
Body fat %		0.75	0.87	0.61	0.9
					
*DXA*					
VAT (cm^3^)		0.94	0.84	0.93	0.82
AF (cm^3^)		0.65	0.97	0.49	0.95

Abbreviations: AF, android fat; BMI, body mass index; DXA, dual-energy X-ray absorptiometry; MRI, magnetic resonance imaging; TAF, total abdominal fat; VAT, visceral adipose tissue.

All correlations had a *P* value <0.0001.

a MRI abdominal fat AF (subcutaneous adipose tissue (SCAT)+VAT).
